# Comparison of cilostazol versus ticlopidine following coronary stenting in patients with coronary heart disease: A meta-analysis of randomized controlled trials

**DOI:** 10.3892/etm.2013.1190

**Published:** 2013-07-01

**Authors:** FENG-HUAN HU, XIN YI, YUE-JING YANG, SHU-BIN QIAO, YONG-JIAN WU, JIAN-SONG YUAN

**Affiliations:** 1 State Key Laboratory of Cardiovascular Disease, Coronary Artery Diseases Center, Fuwai Hospital, National Center for Cardiovascular Diseases, Chinese Academy of Medical Sciences and Peking Union Medical College, Beijing 100037, P.R. China; 2 Department of Cardiovascular Medicine, Beijing Hui People Hospital, Beijing 100054, P.R. China

**Keywords:** coronary heart disease, coronary stenting, cilostazol, ticlopidine, meta-analysis

## Abstract

Previous studies have shown that the combination of cilostazol and aspirin may be a more effective regimen than ticlopidine plus aspirin in the prevention of late restenosis and acute or subacute stent thrombosis following coronary stenting; however, individually published results are inconclusive. The aim of this meta-analysis was to compare the differences in late restenosis and stent thrombosis between cilostazol plus aspirin and ticlopidine plus aspirin for patients with coronary heart disease (CHD) following coronary stenting. A literature search of Pubmed, Embase, Web of Science and Chinese BioMedicine (CBM) databases was conducted from 1998 to March 1, 2013 and statistical analysis was performed using Stata statistical software, version 12.0. Twelve randomized controlled trials were included in the study, with a total of 2,708 patients with CHD following coronary stenting. The patient population comprised 1,371 patients treated with cilostazol plus aspirin and 1,337 patients treated with ticlopidine plus aspirin. The meta-analysis showed that cilostazol plus aspirin demonstrated a lower rate of restenosis than ticlopidine plus aspirin [odds ratio (OR)=0.83, 95% confidence interval (CI)=0.69–0.99, P=0.047]. A significant difference was also observed in the average percent diameter stenosis between cilostazol plus aspirin and ticlopidine plus aspirin [standardized weight difference (SMD)= −0.57, 95% CI=−0.92, −0.23, P=0.001). However, there were no significant differences in the rates of acute or subacute stent thrombosis between cilostazol plus aspirin and ticlopidine plus aspirin. The present meta-analysis suggests that cilostazol plus aspirin may result in a lower restenosis rate and percent diameter stenosis than ticlopidine plus aspirin for patients with CHD following coronary stenting.

## Introduction

Coronary heart disease (CHD), the most common global cause of morbidity and mortality, is known to consume vast medical resources (1,2). Although coronary stenting is widely used in the treatment of patients with CHD, the high rates of late restenosis and stent thrombosis remain the primary limitations (3). At present, adjunctive antiplatelet therapy has been suggested to reduce the incidence rate of restenosis and stent thrombosis (4,5). In addition, the utilization of antiplatelet agents has been demonstrated to be effective in improving final outcomes (6).

Aspirin, a traditional antiplatelet agent, has been most commonly used for the prevention of ischemic arterial events, including coronary thrombosis; however, it has no impact on restenosis (7). Therefore, the introduction of an effective antiplatelet therapy to be used in combination with aspirin and alternative antiplatelet agents following coronary stenting is urgently required. The importance of antiplatelet therapy with ticlopidine plus aspirin in the prevention of subacute thrombosis following coronary artery stenting has been demonstrated (8). However, the use of ticlopidine presents the risk of serious side-effects, such as neutropenia or thrombocytopenia. Cilostazol is a selective cyclic adenosine monophosphate phosphodiesterase inhibitor that is known to inhibit platelet aggregation and intimal hyperplasia (9,10). In view of the fact that cilostazol use presents the risk of mild adverse side effects, cilostazol may theoretically be a desirable substitute for ticlopidine (11). Thus, the adjunctive use of cilostazol plus aspirin following coronary stenting is becoming a more respected option. Recently, it has been demonstrated that cilostazol is able to prevent thrombosis following coronary stenting, reduce restenosis and improve clinical outcomes (12). Moreover, antiplatelet therapy with cilostazol plus aspirin has been shown to be effective in preventing late restenosis and stent thrombosis, with less serious complications (13). However, it has also demonstrated that cilostazol plus aspirin is not able to be statistically distinguished from ticlopidine plus aspirin for the prevention of adverse cardiac events following coronary stenting. Furthermore, a prospective randomized controlled trial revealed that ticlopidine plus aspirin resulted in a significant reduction in subacute thrombosis compared with cilostazol plus aspirin (14).

Therefore, the aim of the present meta-analysis was to compare the differences between cilostazol plus aspirin and ticlopidine plus aspirin with regard to the late restenosis and stent thrombosis rates in patients with CHD following coronary stenting. This may be beneficial in enabling cardiologists to select the anti-platelet therapy method with the greatest efficacy and cost-effectiveness. Furthermore, such knowledge may be further utilized for the accurate determination of treatment strategies for CHD.

## Materials and methods

### Literature search strategy

Relevant papers (published from 1998 to March 1, 2013) were identified through a search in Pubmed, Embase, Web of Science and Chinese BioMedicine (CBM) databases using the following terms: (‘coronary disease’ or ‘coronary diseases’ or ‘disease, coronary’ or ‘coronary heart disease’ or ‘heart disease, coronary’) and (‘stents’ or ‘stent’ or ‘drug-eluting stents’ or ‘bare metal stent’ or ‘percutaneous coronary intervention’) and (‘antiplatelet therapy’ or ‘cilostazol’ or ‘aspirin’ or ‘ticlopidine’). This search strategy was performed iteratively until no other relevant articles were found. The references from the eligible articles or textbooks were also reviewed manually to search for other potential studies. Disagreements were resolved through discussions between the authors.

### Inclusion and exclusion criteria

The inclusion criteria for the studies included in the present meta-analysis comprised: i) randomized controlled trials focusing on the differences in late restenosis and stent thrombosis between cilostazol plus aspirin and ticlopidine plus aspirin for patients with CHD following coronary stenting; ii) studies with follow-up periods of >1 month; iii) studies where the published data concerning the rates of restenosis and stent thrombosis were sufficient; iv) studies published in the English or Chinese languages. Studies were excluded when they were: i) Not clinically-controlled or relevant to the use of cilostazol plus aspirin and ticlopidine plus aspirin for patients with CHD following coronary stenting; ii) duplicates of previous publications; iii) based on incomplete data; iv) case reports, letters, reviews, meta-analyses or editorial articles. If more than one study by the same authors using the same case series was published, either the study with the largest sample size or the most recently published study was selected.

### Data extraction

Using a standardized form, data from the studies were extracted independently by two authors. The following information was obtained for each of the studies: First author, year of publication, country, language, study design, numbers of test subjects, eligible lesions, follow-up periods, antiplatelet drug and dose or dosage, and the rates of restenosis and stent thrombosis. In case of conflicting evaluations, an agreement was reached following a discussion between the authors. When required, a third review resolved any discrepancies or uncertainties with regard to the data extraction process.

### Quality assessment of the included studies

The methodological quality of each of the included studies was evaluated by two independent reviewers using the Physiotherapy Evidence Database (PEDro) scale (15). Eleven assessment items matching with the quality appraisal were used in this meta-analysis, with scores ranging from 0 to 10. The PEDro criteria are based on the presence/absence of 11 items: Eligibility criteria, random allocation, allocation concealment, similar baseline characteristics, blinding of all subjects, blinding of therapists, blinding of outcome assessors, crossover rate of <15%, intention-to-treat analysis, statistical comparisons between groups and measures of variability.

### Statistical analysis

The differences in late restenosis and stent thrombosis rates between cilostazol plus aspirin and ticlopidine plus aspirin were measured by odds ratios (ORs) or standardized weight differences (SMDs), with 95% confidence intervals (CIs). The statistical significance of the pooled value was examined using the Z test. Interstudy variations and heterogeneities were estimated using the Cochran’s Q-statistic, with P<0.05 indicating a statistically significant heterogeneity (16,17). The effect of heterogeneity was also quantified using the I^2^ test (ranges from 0 to 100%), which represented the proportion of interstudy variability that may be contributed to heterogeneity rather than chance. When a significant Q-statistic (P<0.05) or I^2^>50% indicated that heterogeneity existed among the studies, the random effects model (DerSimonian Laird method) was conducted for the meta-analysis; otherwise, the fixed effects model (Mantel-Haenszel method) was used. An analysis of sensitivity was performed by omitting each study in turn to assess the quality and consistency of the results. Begger’s funnel plots were used to detect publication biases. In addition, the Egger’s linear regression test, which measures funnel plot asymmetry using a natural logarithm scale of OR, was used to evaluate the publication biases (18). To ensure the reliability and the accuracy of the results, two authors assessed the data in the statistical software programs independently and obtained the same results. All the P-values were two-sided and all analyses were calculated using Stata statistical software, version 12.0 (Stata Corp., College Station, TX, USA).

## Results

### Characteristics of the included studies

**Figure 1:**
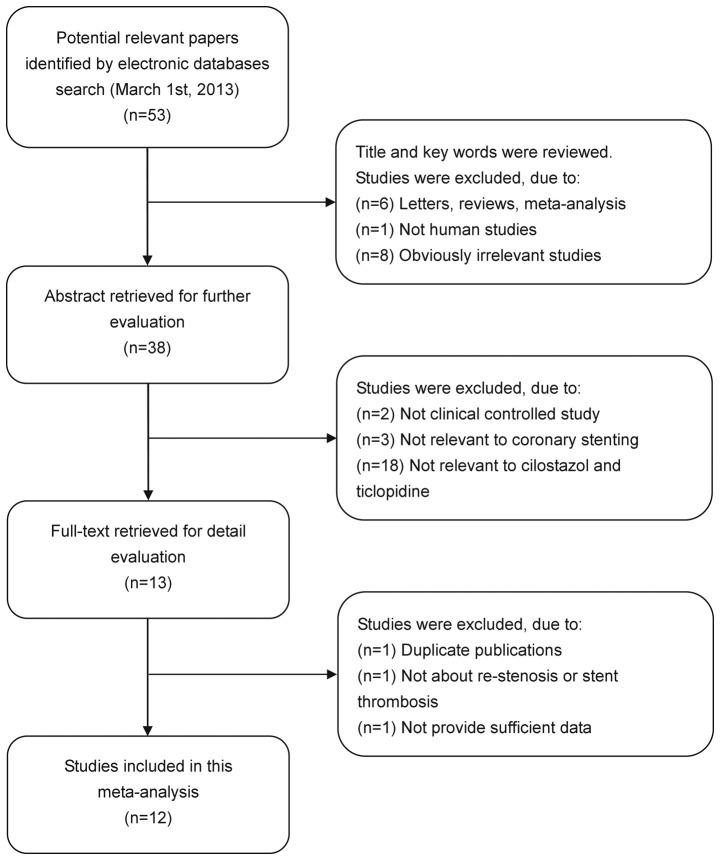
Flow chart of literature search and study selection.

According to the inclusion criteria, 12 randomized controlled trials were included in this meta-analysis (3–5,8,14,19–25). The publication year of the included studies ranged from 1999 to 2006. The flow chart of study selection is shown in Fig. 1. The meta-analysis comprised a total of 2,708 patients with CHD following coronary stenting, including 1,371 patients treated with cilostazol plus aspirin and 1,337 patients treated with ticlopidine plus aspirin. The doses of aspirin ranged from 80 to 243 mg/day, while ticlopidine ranged from 200 to 500 mg/day and cilostazol was administered at 100 mg/bid. The follow-up periods ranged from 1 to 12 months. The main characteristics of all the eligible studies are listed in Table I.

### Quantitative data synthesis

**Figure 2:**
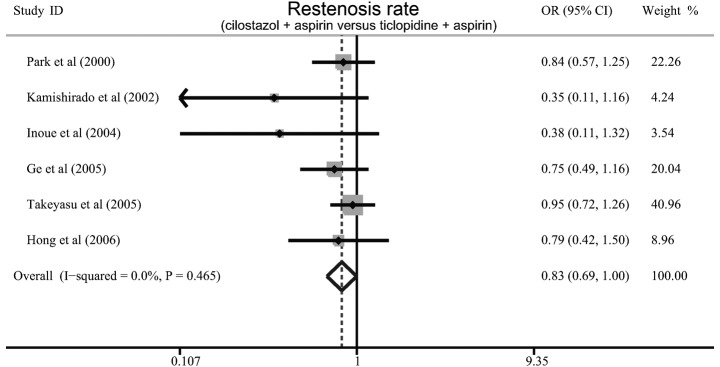
Forest plot of odds ratios (ORs) for the difference in the rate of restenosis between cilostazol plus aspirin and ticlopidine plus aspirin. CI, confidence interval.

**Figure 3:**
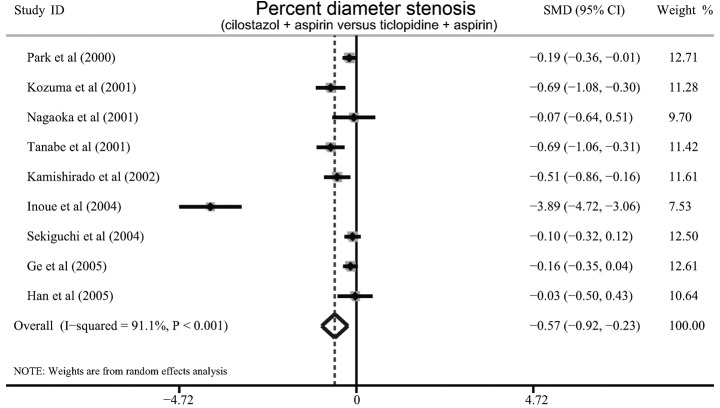
Forest plot of odds ratios (ORs) for the difference in average percent diameter stenosis between cilostazol plus aspirin and ticlopidine plus aspirin. CI, confidence interval.

Six studies referred to the differences between cilostazol plus aspirin and ticlopidine plus aspirin with regard to the rates of restenosis in patients with CHD following coronary stenting. There was no evident heterogeneity (P=0.465, I^2^=0%), and therefore the fixed effects model was used. When all the eligible studies were pooled into the meta-analysis, the results showed that the patients treated with cilostazol plus aspirin exhibited a lower rate of restenosis than those with ticlopidine plus aspirin (OR=0.83, 95% CI=0.69–0.99, P=0.047; Fig. 2). Furthermore, a significant difference was observed in the average percent diameter stenosis between cilostazol plus aspirin and ticlopidine plus aspirin (SMD=−0.57, 95% CI=−0.92 - −0.23, P=0.001; Fig. 3).

**Figure 4:**
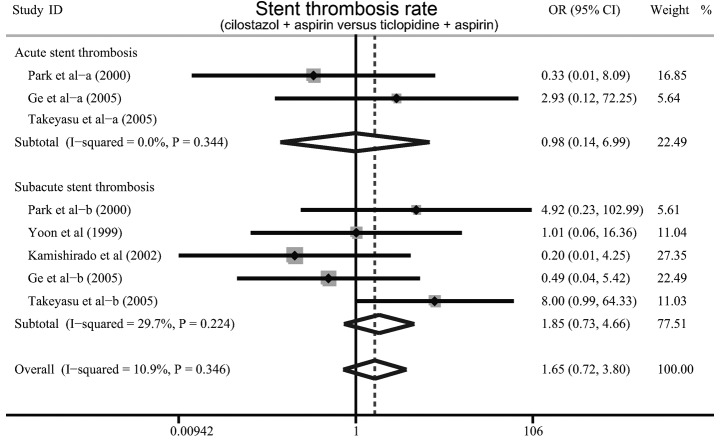
Forest plot of odds ratios (ORs) for the difference in the rates of stent thrombosis between cilostazol plus aspirin and ticlopidine plus aspirin. CI, confidence interval.

The difference in the rate of stent thrombosis between cilostazol plus aspirin and ticlopidine plus aspirin was discussed in six studies. Since no significant heterogeneity was observed, the fixed effects model was used. The results of the meta-analysis showed that the incidence of stent thrombosis in patients treated with cilostazol plus aspirin was not significantly lower than that in those treated with ticlopidine plus aspirin (OR=1.66, 95% CI=0.72–3.80, P=0.235). Furthermore, there were no significant differences in the incidences of acute or subacute stent thrombosis in patients treated with cilostazol plus aspirin compared with those treated with ticlopidine plus aspirin (OR=0.98, 95% CI=0.14–6.99, P=0.983; OR=1.85, 95% CI=0.73–6.99, P=0.467, respectively; Fig. 4).

### Sensitivity analysis and publication bias

**Figure 5:**
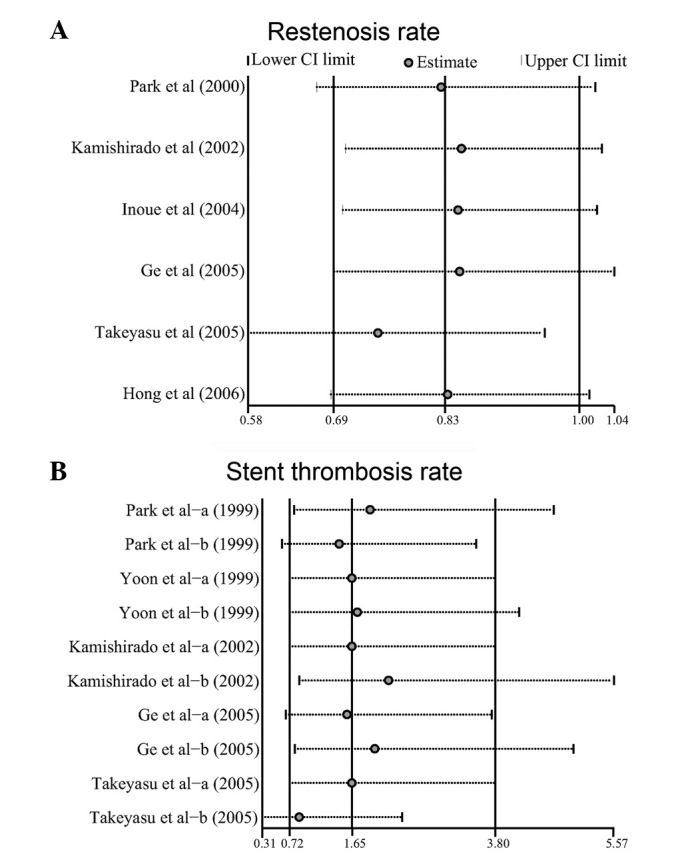
Sensitivity analysis of the summary odds ratio coefficients on (A) the rates of restenosis and (B) stent thrombosis between cilostazol plus aspirin and ticlopidine plus aspirin. ^a^Acute stent thrombosis; ^b^subacute stent thrombosis. Results were computed by omitting each study in turn. Meta-analysis random-effects estimates (exponential form) were used. The two ends of the dotted lines represent the 95% confidence intervals (CIs).

A sensitivity analysis was performed to assess the influence of each individual study on the pooled ORs by omitting each of the individual studies in turn. The analysis results suggested that no individual study significantly affected the pooled values of the rates of restenosis or stent thrombosis (Fig. 5), indicating statistically robust results.

**Figure 6:**
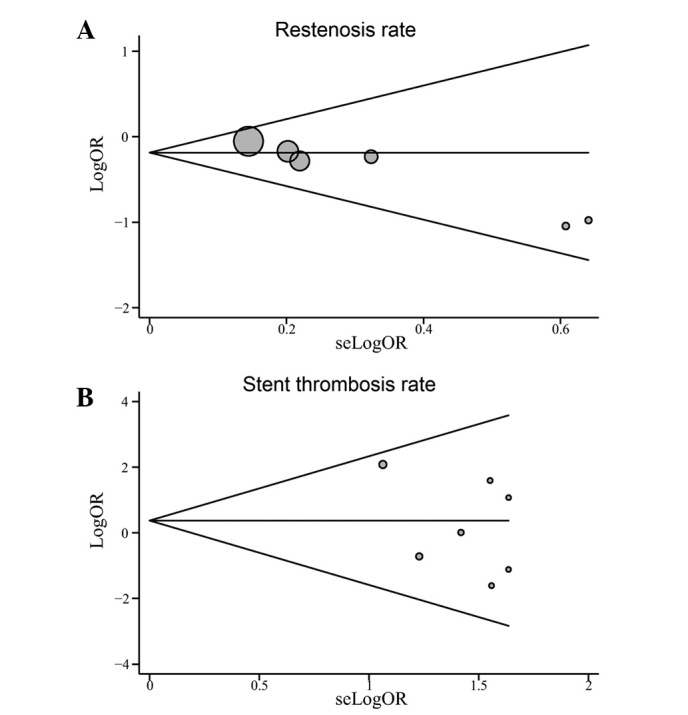
Begger’s funnel plot of publication bias in the selected studies on the rates of (A) restenosis and (B) stent thrombosis between cilostazol plus aspirin and ticlopidine plus aspirin. Each point represents a separate study for the indicated correlation. LogOR, natural logarithm of OR; OR, odds ratio; horizontal line, mean magnitude of the effect; seLogOR, standard error of LogOR.

Publication bias exists to the extent that the available results for a study are unrepresentative of all the results for that study. Begger’s funnel plots and Egger’s linear regression tests were performed to assess the publication bias of the included studies. The shapes of the funnel plots did not reveal any indication of obvious asymmetry (Fig. 6). The Egger’s tests also showed that there was no statistically significant evidence of publication bias for the rates of restenosis and stent thrombosis (t=−2.04, P=0.111; t=−1.18, P=0.292, respectively).

## Discussion

Cilostazol, a selective phosphodiesterase III inhibitor, has been demonstrated to be effective in reducing the incidence of restenosis following coronary stenting (26). At present, the combination of cilostazol and aspirin is regarded as the most acceptable option for the antithrombotic treatment of patients with CHD undergoing coronary stenting (27). However, certain studies have shown that aspirin plus ticlopidine is superior to the combination of cilostazol and aspirin with regard to the midterm occurrence of adverse side-effects (28). Ticlopidine is a potent inhibitor of collagen-induced platelet aggregation, which has been demonstrated to decrease the incidence of clinical events following coronary stenting (14). By activating platelet adenylate cyclase, ticlopidine is able to enhance the stimulatory action of prostaglandin E1 (PGE1) on the cyclase and block the inhibitory action of PGE2 on the cyclase (5). However, compared with cilostazol, the use of ticlopidine may result in more severe side-effects, therefore leading to a shorter course of treatment (29). Numerous studies have been designed to compare the effectiveness of cilostazol plus aspirin with ticlopidine plus aspirin (3,5,21). However, the definite outcomes of the quantitative angiographic analyses of the two antithrombotic regimens remain in dispute. Thus, there is a requirement for a comprehensive well-defined comparison of the two groups of antithrombotic regimens to be implemented. As a powerful statistical method, a meta-analysis provides a quantitative approach for pooling the results of different studies on the same topic. Therefore, a systematic review and meta-analysis of the differences between cilostazol plus aspirin and ticlopidine plus aspirin, with regard to the rates of restenosis and stent thrombosis, was of great value.

In this meta-analysis, 12 randomized controlled studies were included with a total of 2,708 patients with CHD following coronary stenting. The patient population comprised 1,371 patients treated with cilostazol plus aspirin and 1,337 patients treated with ticlopidine plus aspirin. The predominant finding of this meta-analysis was that the rates of restenosis in patients treated with cilostazol plus aspirin were significantly lower than those in patients treated with ticlopidine plus aspirin, suggesting that cilostazol may be more effective than ticlopidine in reducing restenosis. A possible reason may be the different functional mechanisms of the two antithrombotic agents. However, no significant differences were observed in the rates of acute or subacute stent thrombosis between cilostazol plus aspirin and ticlopidine plus aspirin. These results suggested that there was no difference between cilostazol and ticlopidine with regard to their efficacy as an adjunctive therapy to coronary stenting or in the prevention of stent-associated thrombosis. This is despite the fact that cilostazol demonstrates a different anti-platelet mechanism to ticlopidine, which may lead to a suppression of platelet aggregation. These results were inconsistent with the outcomes published by Hashiguchi *et al* and Schleinitz *et al*(6,29), which may be due to the limited number of included studies.

In the interpretation of the results of the present meta-analysis, it is necessary for certain specific issues pertinent to the study to be addressed. The sample size included in the meta-analysis is relatively small and may not provide sufficient statistical power to estimate the differences between cilostazol plus aspirin and ticlopidine plus aspirin. Furthermore, potential heterogeneity and bias may exist due to the differences in the inclusion criteria, follow-up periods, doses of antiplatelet drugs and the severity of disease. In addition, as previously mentioned, each pretreatment regimen was not always identical and the doses of aspirin, ticlopidine or cilostazol were variable. Further limitations included the facts that the type of stent used in each patient was not always identical and there may have been differences with regard to the efficacy of the antiplatelet agents. Moreover, although all participants of each study were well defined with similar inclusion criteria, there may be factors that were not taken into account and that may have influenced our results. There is thus a requirement for the present results to be interpreted with caution due to the potential heterogeneity among trials.

In conclusion, this meta-analysis suggests that the use of cilostazol plus aspirin may result in lower restenosis rates and percent diameter stenosis than ticlopidine plus aspirin for patients with CHD following coronary stenting. However, further well-designed clinical trials are required to investigate the differences between cilostazol and ticlopidine.

## Figures and Tables

**Table I:** Characteristics of included studies in this meta-analysis.

First author (ref)	Year	Country	Number of cases		Follow-up (months)	Drug doses (mg/dosage frequency)		PEDro score
	
			Cilostazol plus aspirin	Ticlopidine plus aspirin		Cilostazol plus aspirin	Ticlopidine plus aspirin	
Yoon *et al*(25)	1999	Korea	147	149	1	aspirin 100 (mg/day)	aspirin 100 mg/day	5
						cilostazol 100 (mg/bid), 1 month	ticlopidine 250 mg/bid	
Park *et al*(23)	2000	Korea	208	201	6	aspirin 200 (mg/day)	aspirin 200 mg/day	6
						cilostazol 100 (bid)	ticlopidine 250 mg/bid	
Kozuma *et al*(22)	2001	Japan	65	65	12	aspirin 200 mg/day	aspirin 200 mg/day	7
						cilostazol 200 mg/day	ticlopidine 200 mg/bid	
Nagaoka *et al*(5)	2001	Japan	18	17	4	aspirin 81 mg/day	aspirin 81 mg/day	6
						cilostazol 200 mg/day	ticlopidine 200 mg/bid, 4 months	
Tanabe *et al*(8)	2001	Japan	54	50	6	aspirin 81 mg/day	aspirin 243 mg/day	5
						cilostazol 200 mg/day	ticlopidine 200 mg/bid	
Kamishirado *et al*(21)	2002	Japan	54	57	6	aspirin 81 mg/day	aspirin 81 mg/day	8
						cilostazol 200 mg/day	ticlopidine 200 mg/bid	
Inoue *et al*(20)	2004	Japan	34	32	-	aspirin 81 mg/day	aspirin 81 mg/day	6
						cilostazol 200 mg/day	ticlopidine 200 mg/bid	
Sekiguchi *et al*(14)	2004	Japan	144	138	6	aspirin 81 mg/day	aspirin 81 mg/day	7
						cilostazol 200 mg/day	ticlopidine 200 mg/bid	
Ge *et al*(3)	2005	China	201	196	9	aspirin 100 mg/day	aspirin 100 mg/day	8
						cilostazol 100 mg/bid	ticlopidine 250 mg/bid	
Han *et al*(4)	2005	China	50	50	6	aspirin 100 mg/day	aspirin 100 mg/day	6
						cilostazol 100 mg/bid	ticlopidine 250 mg/bid	
Takeyasu *et al*(24)	2005	Japan	321	321	6	aspirin 80–200 mg/day	aspirin 80–200 mg/day	6
						cilostazol 200 mg/day	ticlopidine 200 mg/day	
Hong *et al*(19)	2006	China	75	61	6	aspirin 100 mg/day	aspirin 100 mg/day	8
						cilostazol 200 mg/day	ticlopidine 500 mg/day, 1 month	

Ref, reference number; PEDro, Physiotherapy Evidence Database.
